# Acknowledgement to Reviewers of Journal of Cardiovascular Development and Disease in 2019

**DOI:** 10.3390/jcdd7010007

**Published:** 2020-02-06

**Authors:** JCDD Editorial Office

**Affiliations:** MDPI AG, St. Alban-Anlage 66, 4052 Basel, Switzerland

Peer review is an essential part in the publication process, ensuring that Journal of Cardiovascular Development and Disease maintains high quality standards for its published papers. In 2019, a total of 40 papers were published in the journal. Thanks to the cooperation of our reviewers, the median time to first decision was 22.28 days and median APT was 45 days. The editors would like to express their sincere gratitude to the following reviewers for their time and dedication in 2019:
Adhikari, NeetaLin, WenAnderson, RobertLincoln, JoyAnderson, RobertLindeman, JanAngelini, PaoloLiu, ZiqingArbo, IngeridLui, KathyAronsen, Jan MagnusMarino, BrunoBaldini, AntonioMcDaneld, Tara G.Bierhoff, HolgerMistiaen, WilhelmBraun, ThomasMohamed, Salah A.Campione, MarinaMommersteeg, MathildaCampione, MarinaMoore-Morris, ThomasCampione, Marina Naya, FranciscoChaudhry, BillNorris, RussellCherlin, SvetlanaOng, Sang BingColussi, Gian LucaPapazafiropoulou, AthanasiaColussi, Gian LucaPasumarthi, KishoreCooley, Brian C.Pekkan, KeremCorrêa Souza, Gabriela CorrêaPoelmann, RobertDas, SadhanQuax, PaulDe Geest, Bart DeRakowski, TomaszDoppler, StefanieRobbins, JeffreyDzobo, KevinRodríguez, IsabelEl Ghoch, Marwan ElRugonyi, SandraFrangogiannis, NikolaosRugonyi, SandraGarrity, DeborahRugonyi, SandraGrossini, ElenaSardu, CelestinoHalade, GaneshSatou, RyousukeHarper, AlanSchlüter, Klaus-DieterHollander, John M.Sedmera, DavidHosick, PeterSenbonmatsu, TakaakiHu, XiaoyuSerralheiro, PedroHu, XiaoyuSeverino, PaoloHuang, Sarah XuelianShah, Rachana D.Itani, LeilaShiratori, HidetakaIvanovitch, Kenzo D.Stanojevic, Dragana M.Jackson, Alun C.Staples, AnneJaime, Diego FrancoStrychalski, WandaJay, Patrick Y.Tappia, ParamjitJensen, BjarkeThuzar, MoeJokinen, EeroTurner, Neil A.Jouk, Pierre SimonVan Den Hoff, MauriceKing, Benjamin L.Van Eys, Guillaume J.Leung, AlanWeninger, Wolfgang J.Levick, ScottZhao, Jie JaneLevick, ScottZimmermann, WolframLiang, YanZuhl, MicahLiang, Yan


